# Cannabinoid-Induced Immunomodulation during Viral Infections: A Focus on Mitochondria

**DOI:** 10.3390/v12080875

**Published:** 2020-08-11

**Authors:** Cherifa Beji, Hamza Loucif, Roman Telittchenko, David Olagnier, Xavier Dagenais-Lussier, Julien van Grevenynghe

**Affiliations:** 1 Institut National de la Recherche Scientifique (INRS)-Armand-Frappier Santé Biotechnologie, 531 Boulevard des Prairies, Laval, QC H7V 1B7, Canada; cherifa.beji@iaf.inrs.ca (C.B.); hamza.loucif@iaf.inrs.ca (H.L.); roman.telittchenko@iaf.inrs.ca (R.T.); 2 Research Center for Innate Immunology, Department of Biomedicine, Aarhus University, 8000 Aarhus C, Denmark; olagnier@biomed.au.dk

**Keywords:** cannabinoid, THC, mitochondria, immunity, metabolism, inflammation, viral infection, nervous system

## Abstract

This review examines the impact of cannabinoids on viral infections, as well as its effects on the mitochondria of the nervous and immune system. The paper conveys information about the beneficial and negative impacts of cannabinoids on viral infections, especially HIV-1. These include effects on the inflammatory response as well as neuroprotective effects. We also explore non-apoptotic mitochondrial pathways modulated by the activity of cannabinoids, resulting in modifications to cellular functions. As a large part of the literature derives from studies of the nervous system, we first compile the information related to mitochondrial functions in this system, particularly through the CB1 receptor. Finally, we reflect on how this knowledge could complement what has been demonstrated in the immune system, especially in the context of the CB2 receptor and Ca^2+^ uptake. The overall conclusion of the review is that cannabinoids have the potential to affect a broad range of cell types through mitochondrial modulation, be it through receptor-specific action or not, and that this pathway has a potential implication in cases of viral infection.

## 1. Introduction: Importance of Mitochondria on Immunity and Viral Control

Cells can metabolize a variety of carbon substrates including glucose, fatty acids, ketone bodies and amino acids. The cell’s ability to use different carbon sources, which is done through proper mitochondrial function, is critical to their ability to adapt to stress conditions, such as changes in nutrient availability and metabolical needs [1,2,3]. This is especially true in the case of T-cells, where adaptive immunity is dictated by the mitochondria’s capacity to use all types of carbon sources available [4,5]. As an example, naïve T-cells preferentially use oxidative phosphorylation (OXPHOS) in their quiescent state [6], while they switch to glycolysis during their activation [7,8,9]. Although primarily relying on glucose, activated lymphocytes can use many amino acids, such as alanine, leucine, glutamine, serine and arginine, as carbon sources [10,11,12]. For instance, removing alanine and leucine as carbon sources reduces T-cells effector functions and growth [11,12]. Additionally, L-arginine contributes to T-cells’ long-term memory maintenance in the context of OVA immunization in mice [10]. Furthermore, increased mitochondrial fatty acid oxidation (FAO) characterizes memory and regulatory T-cells’ subsets [13,14,15]. Which pathway the mitochondria skews towards is not only important for cell differentiation, it is also critical for proper specific anti-viral response. For example, it is known that polyfunctional anti-human immunodeficiency viruses (HIV-1) CD8 T-cells are critical to control HIV-1 [16,17], and recent evidence shows that the capacity of CD8 T-cells to maintain polyfunctionality in glucose-deprived media might explain natural control of HIV-1 infection [18]. This was partially explained by an increase in lipid uptake. Similarly, CD8 T-cells from controller macaques can target simian immunodeficiency virus (SIV)-infected CD4 T-cells even in the absence of glucose [18]. Additionally, autophagy seems to be important for viral containment and for specific CD8 T-cells’ activity by delivering lipid substrates as an additional energy source [19,20]. HIV, as well as other chronic disease patients such as cancer and sclerosis, have been known to be treated by anti-palliative substances, including cannabis. This plant has a history of being used for therapeutic purposes, namely to alleviate nausea, help calm severe pain, and stimulate the appetite to counter weight loss [21,22,23]. Cannabinoids are a class of compounds that can be found in the cannabis plant. These include a variety of exogenous, endogenous, and synthetic components which exhibit similar psycho-active and immune properties. With their uses in the treatment of advanced stages of illnesses as well as other therapeutic approaches, e.g., as an antidepressant, along with its appetite-stimulating properties becoming more widespread, evidence emerged showing that cannabinoids could influence viral pathogenesis and the immune system [24,25,26,27,28]. Currently, with the increasing number of countries and states legalizing marijuana for recreational use, its impact on health in the context of disease susceptibility is an important aspect to evaluate. The biological activity of cannabinoids is mainly mediated by cannabinoid receptors CB1 and CB2, which are predominantly expressed in cells from the nervous system and immune-derived cells, respectively [29]. When it comes to the immune system, different aspects of it are known to be affected by cannabinoids such as the level of apoptosis, suppression of cell proliferation, inhibition of pro-inflammatory cytokine and chemokine production, and the induction of anti-inflammatory cytokines and regulatory T-cells. In terms of immunomodulation, increasing evidence points towards the cannabinoids-induced effect on metabolism as a crucial player, as it induces AMP-activated protein kinase (AMPK) activation [30,31,32]. Considering the importance of the mitochondria and cellular metabolism for cell function and antiviral response, as mentioned earlier [33], evaluating the role of cannabinoids on both phenomena is important. As such, we wish to place the potential impact of cannabinoids on viral infections, especially in the context of HIV-1, as well as its impact on mitochondria, in the context of the function of the immune system.

## 2. Cannabinoids and Viral Infections: Potential Role of Mitochondria

The effects of cannabinoids on infections, both viral and other types, have already been thoroughly explored in past reviews [24,34,35]. In general, the effect of cannabinoids can be beneficial or detrimental depending on the concentration used, the type of infection, and, of course, which receptor is targeted. Some examples of detrimental effects include: the suppression of inflammatory myeloid cell responses by THC during the response to influenza [36] and the promotion of hepatitis C virus (HCV) replication caused by glucose metabolism disorders of hepatocytes following CB1 activation [37]. In the context of antigen-specific responses, the effects of cannabinoids are still open to question. As an example, it was reported that treatments with THC led to an earlier disease onset in the context of smallpox vaccine virus exposition in mice [38], while in the context of HIV-1, THC enhances antigen-specific immune responses [39]. This study was based on a HIV gp120-derived peptide activation of the immune system. The assessment of the modulating effect of THC was done through the evaluation of antigen-specific responses in both lymphocyte populations as well as non-lymphocyte immune cells in WT and CB1 and CB2 knocked-out mice. For T-cells, peptide-specific activation was determined by the subsequent production of interleukin (IL)-2 and interferon (IFN)-γ, while B-cell activation was studied through the quantification of antibodies production and expression levels of activation markers. On the other hand, a slew of studies report beneficial effects for different treatment procedures such as the attenuation of CXCR4-tropic HIV infection following CB2 activation [40] as well as protection from HIV-1-Tat-mediated neuronal death [41,42] and HIV-1 gp120-mediated neural damage [43]. In this last study, Hu et al. used a human mesencephalic neuronal glial model with a composition similar to the human nervous system, then exposed it to gp120 and finally treated it with THC in a concentration and time of exposure optimized in their previous study. A CB1 and CB2 agonist, WIN55,212-2, significantly reduced the gp120-induced apoptosis in dopaminergic neurons by controlling its subsequent ROS production in microglia. Interestingly, the beneficial effects of cannabinoids on the nervous system are not only found in the case of HIV-1 infection, but also with Theiler’s virus, which is specifically used as a model of demyelination [44,45]. As mentioned earlier, cannabinoids can affect the inflammatory response, which is detrimental in the case of influenza [36]. However, considering that the inflammatory response can be detrimental in some infections [46], this effect of cannabinoids can potentially be beneficial. This is especially true in the context of HIV-1 infection, considering the lingering inflammation present in patients, even under highly active antiretroviral therapy (HAART) [47]. Reports by Henriquez et al. point toward THC as a way to control this inflammation and immune activation, as they demonstrate that treatment with THC reduces both the secretion of IFN-α by plasmacytoid dendritic cells (pDC) and its subsequent activation of T-cells [48,49]. This anti-inflammatory property of THC was also reported in a SIV model, where it reduced T-cell proliferation and activation in the intestine [50]. Similarly, Tahamtan et al. bring forth the important role of the endocannabinoid system in regulating the inflammatory response in the context of respiratory syncytial virus (RSV). In their studies, they demonstrate that activating either CB1 or CB2 decreased immune cell influx and cytokine production, resulting in alleviated lung pathology in a murine model [51,52]. This is particularly interesting considering that supressing inflammation in chronic infections such as HIV-1 is expected to be beneficial in controlling the disease progression since it is mediated through prolonged dysregulation of multiple signalling pathways caused by said inflammation. However, we see how dampening the immune response can also be beneficial in more acute infections, such as RSV (see Table 1 for an overview of the reported effects). The other side of the coin is how viral infections affect the mitochondria. T-cells specific to HIV-1 are subjected to constant immune activation which creates a high energetic demand for those cells [53,54]. Considering this, CD8 T-cells from HIV-1-infected patients not only display an increase in glycolysis but are dependent on glucose for their effector function, even following successful HAART [18]. Additionally, during HIV-1 infection, T-cells become progressively exhausted [55,56], displaying reduced mitochondrial mass and membrane potential. Those phenotypes could be mediated by programmed cell death protein 1 (PD-1) stimulation, resulting in reduced mitochondrial and glycolytic activities [57]. Moreover, another aspect to consider is the effect of treatment on mitochondria health. As an example, nucleoside reverse transcriptase inhibitor (NRTI) treatments for HIV-1 infection have been associated with mitochondrial DNA loss, altered membrane potential, inhibition of electron transport chain complexes, impairment of FAO and lower ATP production [58,59]. Similarly, many reports exist of different viruses modulating the mitochondria to enhance their replication [60,61,62]. Mitochondrial modulations by viruses are twofold. Firstly, considering that mitochondria play a crucial role in antiviral immunity, certain viruses interfere with functions conferring that role in order to proliferate. These include the disturbance of mitochondrial dynamics such as fission and fusion, the induction of mitophagy, and the regulation of apoptotic processes [61]. Secondly, some viruses will exploit the cell’s membrane transport pathways, generating organelles named viral factories. These organelles represent specialized compartments for viral replication, maturation, and export [62]. Given this fact and the role of cannabinoid signalling in modulating the mitochondria, the potential for those compounds to affect viral infections at that level are high, especially considering the reported impact of the endocannabinoid system on HCV replication through its effect on glucose metabolism [37]. However, we have to remain on the side of conjectures, as there are no direct reports on how the mitochondrial effect of cannabinoids might impact viral infections by using such mitochondrial changes. Nevertheless, in the following parts of this review, we will address the impact of cannabinoids on mitochondrial functions of cells from both the nervous and immune system. We explore this side of cannabinoids because, as we will see, they have a wide range of effects, making them strong candidates to control viral infections through mitochondrial modulation.

## 3. Cannabinoids, Mitochondria, and the Nervous System

As cannabinoids have been firstly identified as the neurotropic agents in cannabis, it comes as no surprise that a lot of early studies regarding its mechanism of action were focused on the nervous system. As such, it was discovered that the effects of cannabinoids in the brain are mainly due to the activation of the CB1 receptor. Of note, most of the information concerning the impact of cannabinoids on mitochondria comes from studies in this field of research. As such, even though the main point of this review is to consider the impact of cannabinoids on the immune system, it is necessary to place our knowledge in its initial context: the nervous system. The key element to placing CB1 and mitochondria together comes from the work of Hebert-Chatelain et al., who not only showed the presence of CB1 on the mitochondrial membrane of mouse neurons [79], but also reported its role in the regulation of cellular respiration and energy production [80]. To summarize, they demonstrated that cannabinoid signalling is necessary for PKA-dependent phosphorylation of the mitochondrial electron transport system. Thus, CB1 genetic exclusion leads to decreased cellular respiration. Although those findings came with their share of controversies [81,82], there is now a well-established link between cannabinoids, mitochondria and neuronal activity [83,84,85]. However, Jimenez-Blasco et al. describe in a recent study how the activation of mitochondrial CB1 actually reduces OXPHOS and hampers the metabolism of glucose in mouse astroglia [86]. In this study, they first confirmed that mitochondrial CB1 was responsible for reducing microglia oxygen consumption by comparing the effect of HU210, a CB1 agonist, with that of a cell-impermeable biotinylated version. Mitochondrial CB1 activation then leads to the destabilization of complex I. In addition to the effect on OXPHOS, mitochondrial CB1 activation also leads to a decrease in nuclear hypoxia-inducible factors 1 (HIF-1) resulting in the reduction in glycolytic activity [86]. To summarize, both complete exclusion of mitochondrial CB1 and a strong activation of this same receptor lead to reduced cellular respiration [80,86]. Interestingly, another recent study places CB1 as an important regulator of mitophagy in hippocampal neurons [87], showing that adult hippocampal CB1-KO mice displayed mitochondrial elongation and had reduced mitophagy activity compared to WT. The effect of CB1 knock-out on mitophagy was observed via the levels of Serine 65-phosphorylated ubiquitin serving as a biomarker for PTEN-induced kinase 1 (PINK1) activity. However, Kataoka et al. did not consider the localization of the CB1 to contextualize their observation. In addition to CB1-mediated mitochondrial modulation, Fisar et al. also demonstrated a non-receptor mechanism. They came to this conclusion after evaluating the effects of different cannabinoids with known receptor targets. Overall, they posit that cannabinoids can accumulate in the hydrophobic parts of the inner mitochondrial membrane, impairing the molecular interactions and assembly of the respiratory chain [88]. This comes in addition to previous reports of non-receptor specific effect of cannabinoids on mitochondria [89,90]. Interestingly, it has been shown that the CB1-mediated effect on mitochondrial dynamics is not restricted to cells of the nervous system [91]. In this study, it is shown that CB1 stimulation by the endocannabinoid, n-arachidonoylethanolamine (AEA), in renal proximal tubular cells leads to dynamin-related protein 1 (DRP1) activation, with its subsequent translocation to the mitochondria that results in mitochondrial fission. Inevitably, looking at the impact of cannabinoids on the nervous system leads to having a look at the microglia. These are specialized macrophages found in the central nervous system (CNS). They are important for maintaining the health of the CNS by dealing with infections and removing damaged neurons [92]. In this context, Ma et al. found that AM1241, a CB2 agonist, mediates anti-inflammatory responses in microglia. This was observed by the upregulation of markers associated with M2 phenotypes, such as arginase 1 and brain-derived neurotrophic factor with a downregulation of the M1 markers’ inducible nitric oxide synthase and tumor necrosis factor (TNF)-α. They suggest that this might be due to the peroxisome proliferator-activated receptor gamma coactivator 1-alpha (PGC-1α) and its association with the enhancement of mitochondria biogenesis. [93]. Both the idea that the effect of cannabinoids on mitochondria is not specific to neuronal cells as well as having a direct impact on microglia, which bridges the nervous and immune system, leads us to review the impact of cannabinoids on the mitochondria of immune cells.

## 4. Cannabinoids, Mitochondria, and the Immune System

**Figure 1:**
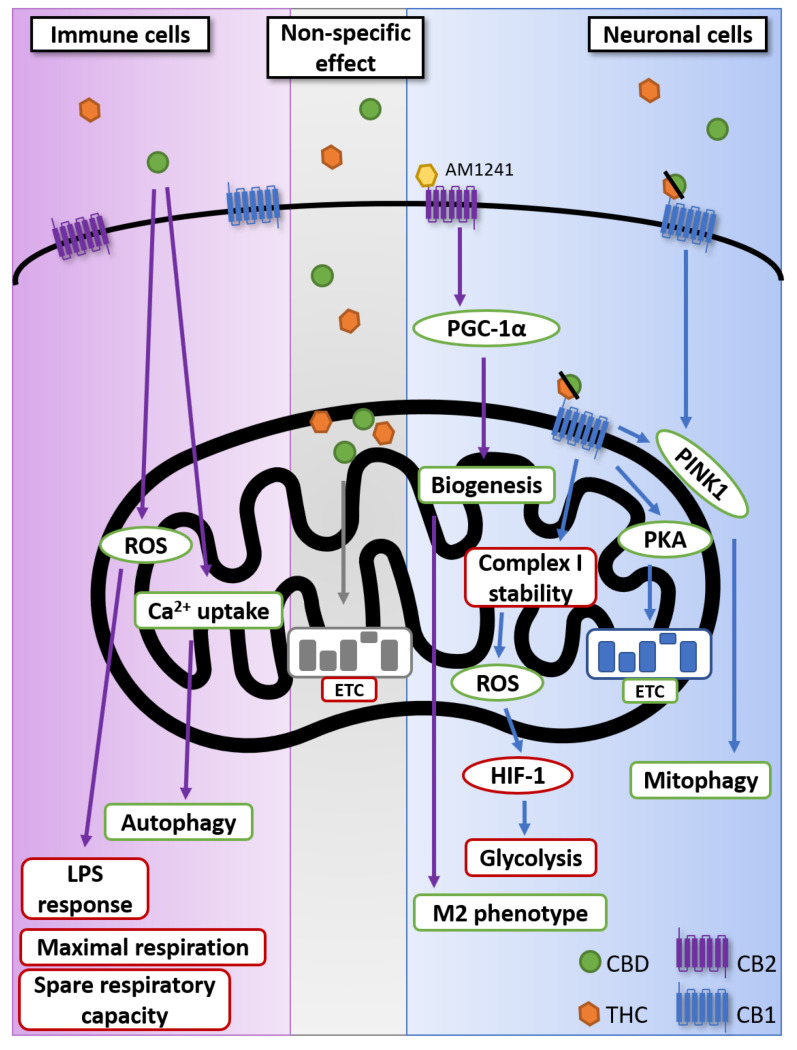
Non-apoptotic impact of cannabinoids on mitochondria. Although CB1 and CB2 are found on immune cell, the reported activity of CBD on the mitochondria of immune cells was not confirmed to be dependent on either receptor [95,100,103]. Non-specific effect represents the effect of cannabinoids on mitochondrial membrane integrity demonstrated not to be dependent on CB1 or CB2 [88,89,90]. It is now well established that CB1 is found on mitochondrial membrane [80,86,87,93]. Green borders represent an upregulation or increased activity; red borders represent a downregulation or reduced activity. CB1: Cannabinoid receptor type 1; CB2: Cannabinoid receptor type 2; CBD: Cannabidiol; ETC: Electron transport chain; HIF-1; hypoxia-inducible factors 1; PGC-1α: peroxisome proliferator-activated receptor gamma coactivator 1-alpha; PINK1: PTEN-induced kinase 1; PKA: Protein kinase A; ROS: reactive oxygen species; THC: Δ⁹-tetrahydrocannabinol.

CB2, being abundantly present on immune cells, has made the study of cannabinoids in the context of infection a necessity. It is now well established that the activation of CB2 receptors, by endogenous or exogenous cannabinoids, is immunosuppressive [26,27]. A recent review is particularly thorough in describing the effects of cannabidiol (CBD) in regulating the immune responses [94] In the case of T-cells, this is notable through their suppression of IL-2, IFN-γ, and TNF-α [95]. Considering the crucial role that the mitochondria plays in apoptosis as well as in various intrinsic and extrinsic ways [96,97], it comes as no surprise that most studies of cannabinoids on mitochondria revolve around apoptosis [98]. One of the most recent examples of this is a study by Wu et al., who showed that CBD had a very important pro-apoptotic effect on monocytes because of its effect on mitochondrial membrane potential and cytochrome c release [99]. Although the studies indicating the non-apoptotic effect of cannabinoids on the mitochondria from immune cells remain few, some point towards a role in reactive oxygen species (ROS) production and an effect on mitochondrial respiration [95,100]. In their paper, Schultze et al. demonstrate that CBD reduces both maximal respiration and spare respiratory capacity in THP-1 monocytes [100]. Additionally, mitochondrial ROS could potentially have a role to play in LPS-induced inflammatory responses by macrophages. In this study, a 24 h treatment with CBD dramatically reduced IL-8 production by U937 monocytes [95]. Although in both studies they observed an increase in mitochondrial ROS, the effects observed were not directly linked to its production. Additional studies, with antioxidants, for example, are warranted to elucidate the role of ROS in the non-apoptotic effect of CBD on the mitochondria. As mentioned, the study of the impact of cannabinoids on the metabolism of mitochondria of immune cells is still at an early stage, especially in cells other than myeloid cells. Still, with the evidence currently at our disposition, we can see the potential importance of the cannabinoid pathway in the regulation of immune cell metabolism. Another potential non-apoptotic pathway to modulate mitochondria by cannabinoids is through calcium signalling. Indeed, ion channels, especially calcium, are known to be crucial for proper immune responses [101,102]. In this context, Olivas-Aguirre et al. found that CBD favors mitochondrial Ca^2+^ uptake on acute lymphoblastic leukemia of T lineage cells. This leads to a loss of mitochondrial membrane potential, disruption of the cristae, and ATP production, ultimately resulting in mitophagy. At sublethal concentration, they demonstrate that CBD induces a conversion of LC3-I to LC3-II, showing an activation of autophagy. [103]. This is of particular interest considering that Ca^2+^ intake is required for T-cell receptor stimulation [102,104]. Overall, the impact of cannabinoids on non-apoptotic mitochondrial pathways remains to be fully uncovered, but with the current knowledge at our disposition, we can expect it to have important ramifications for the immune system regulation, particularly in the inflammatory responses (see Figure 1 for an overview of non-apoptotic effect of cannabinoids on mitochondrial functions).

## 5. Conclusions

**Figure 2:**
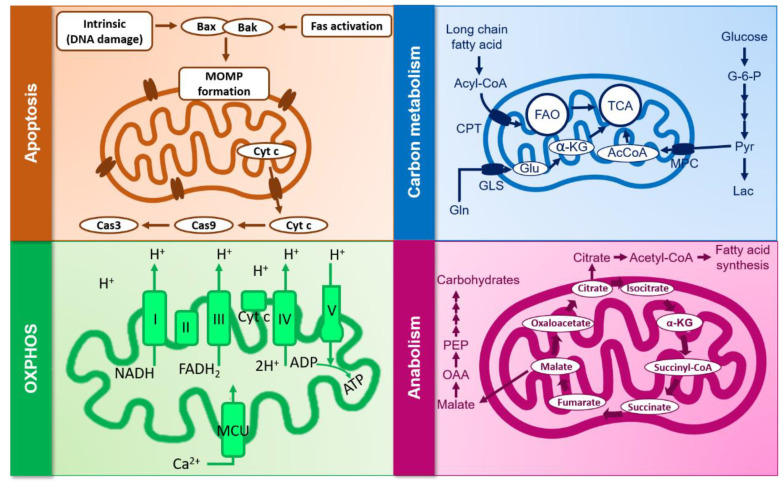
Mitochondrial processes potentially affected by cannabinoids. α-KG: alpha-ketoglutarate; CPT: carnitine palmitoyltransferase; FAO: fatty acid oxidation; GLS: mitochondrial glutaminase; MOMP: mitochondrial outer membrane permeabilization; MCU: mitochondrial calcium uniporter; MPC: mitochondrial pyruvate carrier; OAA: oxaloacetate; TCA: tricarboxylic acid cycle.

The recent legalization of marijuana in Canada, as well as in different European countries, made cannabinoids accessible as off-the-counter substances for patients in need of palliative treatment, as well as for recreational users. As such, the study of the effects of endogenous as well as exogenous cannabinoids on the immune system, be it in a resting state or in the case of viral infections, becomes crucial. Aside from their apoptotic and non-apoptotic effects, their impact on viral infections as well as inflammation and their effect on cellular metabolism and energy uptake, and sex- and age-dependent differences, in metabolizing such substances have been pointed out. In a study by Fattore et al., although no dissimilarities were pointed out when it comes to intoxication or plasmic THC levels, male smokers showed notably higher levels in circulating THC levels and larger cardiovascular effects than female smokers, who comparatively showed higher CB1 protein expressions and more explicit hemodynamic changes [105]. In line with this study, marijuana metabolizing is also impacted by body fat levels and sexual hormones, particularly testosterone and estrogen, which, respectively, lighten and intensify THC absorption and thus impact its conversion into inactive or active metabolites such as 11-hydroxy-THC [106]. In a similar context, a recent review by Gorey et al. summarized the impact of age on cannabis use and metabolization by focusing on its effect on attention, learning and memory in youths (under 18 years old) and adults (over 18 years old) almost daily on daily smokers as well as in young and adult rats [107]. The paper showed that multiple gaps and contradictions exist in studies done on both human and rats when it comes to THC effect on neuropsychological domains, but that young smokers showed more attention deficit as well as lower prose recall compared to adults, who showed higher memory impairments. Although the effects of cannabinoids on inflammation are well established, information about how the mitochondria are involved remains limited. Considering all of the facts above, cannabinoids’ metabolism, be it derived from cannabis or synthetic substances, needs to be further investigated, especially when it comes to its effect on mitochondrial plasticity in the context of viral infections and the resulting chronic inflammation. This is notable considering the growing interest in its usage during persistent infections which are associated with inflammation [34,108]. Another aspect to remember is the lipophilic nature of cannabinoids that allows them to enter cells passively. Given this property, they can potentially alter the integrity of any cellular membrane regardless of the presence of CB1/CB2 by accumulating in the hydrophobic parts, leading to impairment of molecular interactions [88]. An innovative strategy to investigate such an effect will be the use, development, and optimization of the Seahorse, which is a novel live-cell metabolic assay platform that allows the real time evaluation of cellular respiration and plasticity via the accurate measurements of mitochondrial respiration and plasticity (see Figure 2 and Table 2 for a summary of mitochondrial processes potentially affected by cannabinoids and in which cell type the effect was reported).

## Figures and Tables

**Table 1:** Effects of cannabinoids on viral infections. 2-AG: 2-Arachidonoylglycerol; AEA: N-arachidonoylethanolamine; CB1: Cannabinoid receptor type 1; CB2: Cannabinoid receptor type 2; CBD: Cannabidiol; HBV: hepatitis B virus; MAIDS: murine acquired immunodeficiency syndrome; THC: Δ⁹-tetrahydrocannabinol.

Virus	Treatment/Context	Model	Observations	Reference
HIV-1				
	THC	Human	Suppression of IFN-α-mediated activation of T-cells	Henriquez et al., 2018 [48]
	Inhibition of AEA hydrolysis	Murine	Reduction in HIV-Tat-mediated neuronal death and dendritic degeneration	Hermes et al., 2018 [41]
	Cannabis use in HAART treated patients	Human	Reduction in systemic inflammation and immune activation	Manuzak et al., 2018 [63]
	AEA, 2-AG	Murine	Protection of neurons from HIV-Tat excitotoxicity	Xu et al., 2017 [42]
	THC	Human	Suppression of IFN-α secretion by pDC	Henriquez et al., 2017 [49]
	THC	Murine	Enhancement of pVRCgp120-induced IFN-γ production	Chen et al., 2015
			by splenic lymphocyte populations and activation of T/B cells	[39]
	AEA, 2-AG	Human	Suppression of pro-inflammatory and increase of anti-inflammatory cytokines, through the MAPK pathway	Krishnan and Chaterjee, 2014 [64]
	THC	Human	Reduction of cell surface HIV receptor (CD4, CCR5 and CXCR4) expression on macrophages	Williams et al., 2014 [65]
	THC, CP55940 (CB1/2 agonist)	Human	Inhibition of HIV-Tat-mediated adhesion of monocyte to extracellular matrix	Raborn et al., 2014 [66]
	JWH133, Gp1a, O-1966 (CB2 agonist)	Human	Inhibition of RT and LTR activity	Ramirez et al., 2013 [67]
	WIN55,212-2 (CB1/2 agonist)	Human	Protection of human dopaminergic neurons from gp120	Hu et al., 2013 [43]
	THC, CBD	Murine	Enhancement of T-cell response after suboptimal stimulation	Chen et al., 2012 [68]
			Suppression of T-cell response after optimal stimulation	
	JWH133, JWH150, 2-AG, AEA (CB2 agonists)	Human	Reduces cell-free and cell-to-cell transmission of CXCR4-tropic HIV	Constantino et al., 2012 [40]
HIV-1	THC, 2-AG	Murine	Reduction CCR3 expression resulting in less migration of BV-2 cells towards HIV-Tat	Fraga et al., 2011 [69]
	WIN55,212-2 (CB1/2 agonist)	Murine	Inhibited gp120-induced IL-1β production and impairment of network functions	Kim et al., 2011 [70]
	THC, CP55940 (CB1/2 agonist)	Human	Inhibition of macrophage migration to HIV-Tat protein	Raborn and Cabral, 2010 [71]
SIV				
	THC	Rhesus	No upregulation of pro-inflammatory miR-21, miR-141 and miR-222 and alpha/beta defensins	Kumar et al., 2019 [50]
			Higher expression of tight junction proteins (occludin, claudin-3), anti-inflammatory MUC13, keratin-8 (stress protection), PROM1 (epithelial proliferation)	
	THC	Rhesus	Upregulation of microRNA which targets proinflammatory molecules	Chandra et al., 2015 [72]
	THC	Rhesus	Chronic administration increased CXCR4 expression on T-cells	LeCapitaine et al., 2011 [73]
	THC	Rhesus	Chronic administration reduced early mortality, associated with attenuation of plasma viral load and body mass retention	Molina et al., 2011 [74]
MAIDS				
	JWH015, JWH133, Gp1a (CB2 agonists)	Murine	Acute antiallodynic effects on infection-induced neuropathic pain	Sheng et al., 2019 [75]
HBV				
	Rimonabant (CB1 inhibitor)	Human	Suppressed HBV propagation through the inhibition of hepatocyte nuclear factor 4α	Sato et al., 2020 [76]
HCV				
	AEA	Human	Decrease of AMPK phosphorylation, inhibition of cell surface expression of GLUT2, and suppression of cellular glucose uptake. Promotion of viral replication	Sun et al., 2014 [37]
RSV				
	JZL184 (CB1 agonist)	Murine	Decreased immune cell influx and cytokine/chemokine production, and alleviated lung pathology	Tahamtan et al., 2018 (a) [51]
	JWH133 (CB2 agonist)	Murine	Decreased immune cell influx and cytokine/chemokine production, and alleviated lung pathology	Tahamtan et al., 2018 (b) [52]
Theiler’s				
	CBD	Murine	Decreased frequency and severity of acute behavioral seizures	Patel et al., 2019 [45]
	Inhibition of 2-AG hydrolysis	Murine	Enhances remyelination	Feliu et al., 2017 [44]
	WIN55,212-2 (CB1/2 agonist)	Murine	Reduced CD4 + CD25 + Foxp3– T-cells activation in the CNS and increased regulatory CD4 + CD25 + Foxp3 + T-cell activation	Arevalo-Martin et al., 2012 [77]
	AEA	Murine	Inhibition of VCAM-1 potentially reducing neuroinflammation	Mestre et al., 2011 [78]
Influenza				
	THC	Murine	Suppressed DC, macrophages, monocytes, and inflammatory myeloid cell responses	Karmaus et al., 2013 [36]
Vaccinia				
	THC	Murine	Increased severity and duration of symptoms	Huemer et al., 2011 [38]

**Table 2:** Mitochondrial processes affected by cannabinoids in neuronal and immune cells. “+” indicates reported increase, “−” indicates reported reduction in the process. Although evidence suggests that carbon metabolism and anabolism might be affected by cannabinoids through AMPK activity, there are no direct reports of those effect [32,86,98,99,100,109,110,111,112].

	Neuronal	Immune
Apoptosis	−	+
Carbon metabolism	−	?
OXPHOS	+/−	−
Anabolism	?	?
